# CT measured pulmonary artery to ascending aorta ratio stratified by echocardiographically obtained systolic pulmonary artery pressure values for noninvasive detection of pulmonary hypertension in patients with severe aortic valve stenosis

**DOI:** 10.1007/s00392-023-02182-8

**Published:** 2023-03-20

**Authors:** Elke Boxhammer, Joseph Kletzer, Jörg Kellermair, Bernhard Scharinger, Reinhard Kaufmann, Matthias Hammerer, Hermann Blessberger, Clemens Steinwender, Michael Lichtenauer, Klaus Hergan, Uta C. Hoppe, Stefan Hecht

**Affiliations:** 1 Department of Internal Medicine II, Division of Cardiology, Paracelsus Medical University of Salzburg, 5020 Salzburg, Austria; 2 Department of Cardiology, Johannes Kepler University Hospital Linz, 4020 Linz, Austria; 3 Department of Radiology, Paracelsus Medical University of Salzburg, Müllner Hauptstraße 48, 5020 Salzburg, Austria

**Keywords:** Aortic valve stenosis, Biomarker, PA/AA-ratio, Pulmonary hypertension, Systolic pulmonary artery pressure

## Abstract

**Background:**

Transthoracic echocardiography (TTE) offers a measurement method for the determination of pulmonary hypertension (PH) in patients with severe aortic valve stenosis (AS) with determination of maximal tricuspid regurgitation velocity (TRVmax) and systolic pulmonary artery pressure (sPAP). Radiological parameters for noninvasive detection of PH, most importantly computed tomography (CT) based PA/AA-ratio = ratio of pulmonary artery diameter (PA) and ascending aorta diameter (AA), are also included in the latest ESC guidelines. The aim of the present study was to define cut-off values for PA/AA-ratio taking also into account cardiovascular biomarkers to determine criteria for noninvasive diagnosis of PH.

**Methods:**

194 patients with severe AS undergoing transcatheter aortic valve replacement (TAVR) underwent pre-procedural TTE and CT with measurement of PA/AA-ratio. Additionally, common cardiovascular biomarkers were determined.

**Results:**

TAVR patients with an sPAP ≥ 40 mmHg or a TRVmax ≥ 2.9 m/s had a PA/AA-ratio ≥ 0.80 in an AUROC analysis. The cut-off value of ≥ 0.80 resulted in a significantly higher mortality rate (log-rank test: p = 0.034) in these patients in a Kaplan–Meier analysis regarding 1-year survival after TAVR. Significant differences in biomarker expression between patients with a PA/AA-ratio ≥ 0.80 or < 0.80 occurred for BNP (p = 0.001), cTnI (p = 0.032), GDF-15 (p = 0.002) and H-FABP (p = 0.015).

**Conclusion:**

PA/AA-ratio ≥ 0.80 is a promising radiological parameter that can provide information about mortality in patients with severe AS undergoing TAVR; combined with biomarkers it may contribute to noninvasive detection of PH in patients with severe AS.

**Graphical abstract:**

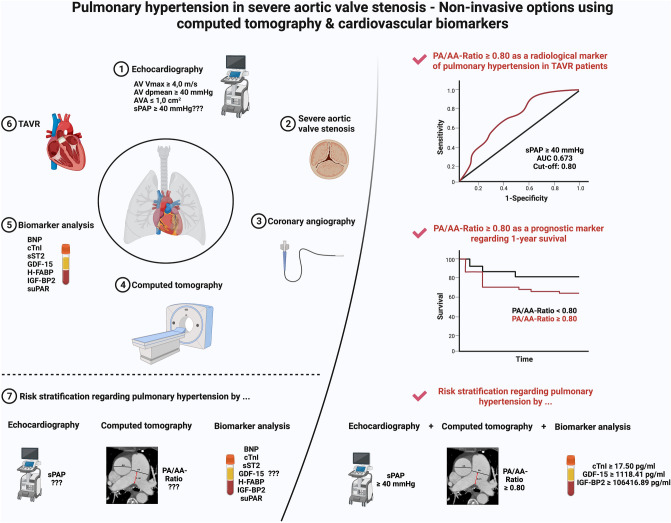

## Introduction

Pulmonary hypertension (PH) due to severe aortic valve stenosis (AS) is a frequently diagnosed sequelae in the clinical setting, which occurs pathophysiologically due to chronic pressure load initially on the left ventricle and subsequently on the left atrium and pulmonary circulation [1]. Clinically, the gold standard of right heart catheterization as a preoperative diagnostic tool has been abandoned and transthoracic echocardiography (TTE) is used as the tool of choice to assess the potential presence of PH in severe AS [2]. The systolic pulmonary artery pressure (sPAP) can be determined and estimated to differentiate between the presence and absence of PH in clinical setting, although various thresholds are used and discussed controversially in the literature. In most cases, a present sPAP ≥ 40 mmHg is considered as relevant pulmonary hypertension [3–5]; however, there are other studies that refer to an sPAP threshold of 45 mmHg [6, 7] or even 50 mmHg [8, 9] in their work. In recent years, however, maximal tricuspid regurgitation velocity (TRVmax) has become increasingly important in addition to sPAP, especially in research setting. A cut-off value of 2.9–3.4 m/s indicates an intermediate probability and > 3.4 m/s indicates a high probability of pulmonary hypertension [10]. Also, TTE is highly dependent on the experience of the corresponding examiner or also on the available sound quality of the subject, so that erroneous measurements or non-conclusive data may occur.

In order to still provide patients with noninvasive options for PH detection, imaging plays an increasingly crucial role. It was already shown in a previous work [11] that the isolated use of a cut-off value of ≥ 29 mm for the main pulmonary artery diameter as defined by the European Society for Cardiology (ESC) guidelines of 2015 [12] is only an estimation and less a solid basis for the presence of PH. In addition, the currently valid ESC guidelines of 2022 [13] describe the ratio of pulmonary artery diameter (PAA) and ascending aorta diameter (AA)—so-called PA/AA-ratio—with a cut-off value of 0.9 as a potentially more accurate and valuable methodology of noninvasive radiological PH determination.

Therefore, the aim of the present study was to calculate a cut-off value for PA/AA-ratio based on echocardiographic sPAP data, to further investigate this value with respect to mortality, and finally to further underline PH determination based on this radiological criterion with serum biomarker analyses of various common cardiovascular biomarkers.

## Material and methods

### Study population

Originally, this study population included 221 patients just before transcatheter aortic valve replacement (TAVR) procedure between 2016 and 2018 at Paracelsus Medical University Hospital Salzburg and Kepler University Hospital Linz. 27 patients had to be excluded due to missing weight or height data, missing CT data or inadaquate CT quality. At last, 194 patients were recommended for inclusion in the study. Relevant exclusion criteria even before study recruitment were patients with a bicuspid aortic valve, acute cardiac decompensation at the time of TTE or at the time of TAVR, as well as patients with any history that might indicate a pre-capillary component of pulmonary hypertension [chronic thrombembolic pulmonary hypertension (CTEPH), idiopathic pumonary arterial hypertension, intersitial lung disease or underlying rheumatologic diseases with pulmonary involvement such as scleroderma, lupus erythematosus, etc.]. Therefore, in this selected patient population, a post-capillary cause [severe AS and possibly limited left ventricular ejection fraction (LVEF)] is assumed for the pulmonary hypertension.

The study protocol was approved by the local ethics committees of Paracelsus Medical University Salzburg (415-E/1969/5-2016) and Johannes Kepler University Linz (E-41-16) and conducted in accordance to principles of the Declaration of Helsinki and Good Clinical Practice. Written informed consent to participate in the study was available from all patients before study inclusion.

### Transthoracic echocardiography

Common ultrasound devices (iE33 and Epiq 5; Philips Healthcare, Hamburg, Germany) were used for performing TTE as routinely diagnostic on average 1–4 weeks before TAVR. These examinations were each conducted by experienced clinicians with more than 4 years of training in echocardiography. Severe AS was classified according to current guidelines of European Society for Cardiology measuring using an AV Vmax (maximal velocity over aortic valve) of 4.0 m/s, an AV dpmean (mean pressure gradient over aortic valve) ≥ 40 mmHg and an aortic valve area ≤ 1.0 cm^2^ for definition of severe AS. Patients with low-flow, low-gradient AS situation were excluded. Via Simpson’s method LVEF was calculated. To graduate mitral, aortic and tricuspid valve regurgitation in minimal, mild (I), moderate (II) and severe (III) spectral and color-Doppler images were used. Maximum tricuspid regurgitant jet velocity (TRVmax) was obtained by continuous wave Doppler over the tricuspid valve. The probability of the presence of PH was considered medium at a threshold of ≥ 2.9 m/s and high at a threshold of ≥ 3.4 m/s. Pulmonary artery pressure (PAP) was calculated using the formula 4 × TRVmax^2^ and adding the estimated right atrial pressure (RAP). The latter corresponds to the central venous pressure and was determined by the diameter of the inferior vena cava (IVC). With an IVC diameter ≥ 21 mm and a respiratory caliber fluctuation < 50%, a RAP of 15 mmHg was assumed. For an IVC diameter < 21 mm as well as a respiratory caliber fluctuation ≥ 50%, a RAP of 3 mmHg was calculated. Other scenarios not corresponding to these constellations were provided with an intermediate value of 8 mmHg [14]. Finally, the simplified Bernoulli Eq. (4 × TRVmax^2^) + RAP was applied to obtain a sPAP result. Different TRVmax (≥ 2.9 m/s and ≥ 3.4 m/s) values and sPAP (40, 45 and 50 mmHg) values were used to determine PH in accordance with the current literature [15–19].

### CTA protocol and measurement of MPA diameter for PH assessment

The included study patients at both centers routinely received a pre-interventional, ECG triggered CTA of the whole aorta and femoral arteries to asses, among others, the aortic annulus size, the aortic anatomy and vascular access. Scans were performed on multidetector CT scanners (Somatom Definition AS+, Siemens Healthcare, Erlangen, Germany; Brilliance 64, Philips Healthcare, Hamburg, Germany) with a patient size-adapted tube voltage (80–120 kVp) and active tube current modulation. A bolus-tracking technique was applied with a 100 mL bolus of non-ionic iodinated contrast media followed by 70 mL saline solution injected at a flow rate of 3.5–5 mL/s. This imaging, as well as TTE, was completed in a separate inpatient stay approximately 1–4 weeks before the TAVR procedure.

**Fig. 1:**
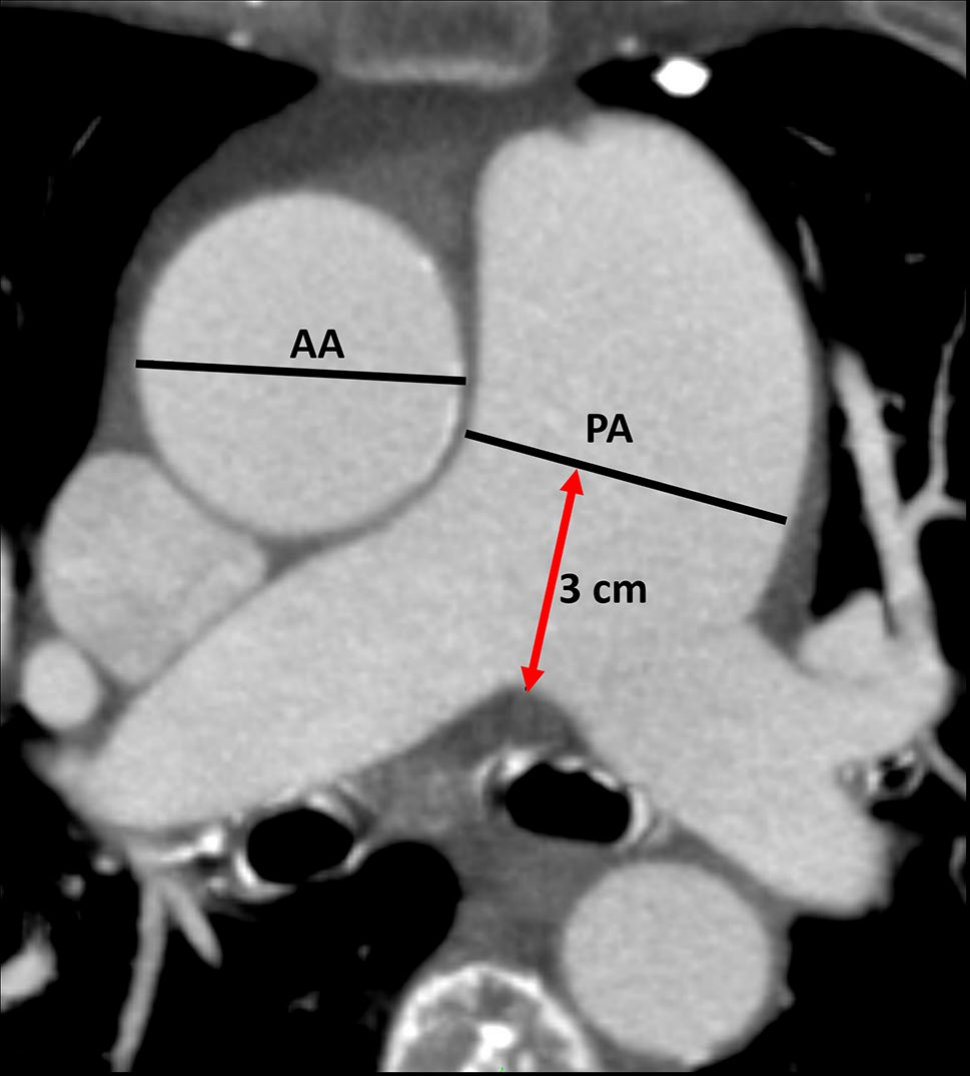
Measurement of diameters of interest on axial CT. AA: ascending aorta; PA: main pulmonary artery; red double headed arrow: distance between bifurcation of the pulmonary trunk and level of measurement within main pulmonary artery

A stationary workstation (Impax, Agfa-Gevaert, Mortsel, Belgium) was used for image analysis. Two experienced radiologists—one board certified with nine years of experience in vascular imaging (radiologist 1), one in the fourth year of training (radiologist 2)—independently performed the following measurements on axial sections in mediastinal window settings on the end-diastolic phase: (1) Main pulmonary artery (PA) diameter was measured perpendicular to the vessel axis at the widest point within 3 cm of the bifurcation of the pulmonary trunk. (2) At the same level as the main PA measurement, the widest diameter of the ascending aorta (AA) was measured (Fig. 1) as previously described [20, 21]. The quotient of main pulmonary artery diameter and ascending aortic diameter formed the PA/AA-ratio as the basis for the CT-based, radiological definition of PH. The results of both observers were compared in terms of inter-observer variability, and the mean value was used for further analysis. 50 randomly selected cases were re-assessed by radiologist 2 after a 4-week interval to evaluate intra-observer variability. The radiologists were blinded to all clinical, hemodynamic and laboratory data.

### Biomarker analysis

Blood samples were taken from the patients in a fasting state and in an upright position using a vacuum-containing system on the day of hospitalization and thus one day before the TAVR procedure. By centrifugation of the collection tubes the plasma was separated from the blood components. Afterwards, plasma was frozen at − 80 °C. All 194 samples were measured at similar time points under same conditions.

Plasma levels of soluble suppression of tumorigenicity-2 (sST2: pg/ml), growth/differentiation of factor-15 (GDF-15: pg/ml), heart-type fatty-acid binding protein (H-FABP: ng/ml), insulin like growth factor binding protein 2 (IGF-BP2: pg/ml) and soluble urokinase-type plasminogen activator receptor (suPAR: pg/ml) were analyzed using enzyme-linked immunosorbent assay (ELISA) kits (sST2: Duoset DY523, GDF-15: Duoset DY957, H-FABP: Duoset DY1678, IGF-BP2: Duoset DY674, suPAR: Duoset DY807, R&D Systems, USA). Manufactures’s instructions were performed for adequate preparation of reagents. Therefore, serum samples and standard protein were placed onto the wells of ELISA plates (Nunc MaxiSorp flat-bottom 96 well plates, VWR International GmbH, Austria) and incubated for 2 h. The plates were treated with Tween 20/PBS solution (Sigma Aldrich, USA). Afterwards, a biotin-labeled antibody was added and incubated for another two hours. A washing process was performed and streptavidin-horesradish-peroxidase solution was added to the wells. A color reaction was generated after adding tetramethylbenzidine (TMB; Sigma Aldrich, USA). Optical density was determined at 450 nm on an ELISA plate-reader (iMark Microplate Absorbance Reader, Bio-Rad Laboratories, Austria).

### Statistical analysis

Statistical analysis was performed using SPSS (Version 25.0, SPSSS Inc., USA). Graphical representations were created using GraphPad Prism (Version 8.0.0, GraphPad Software, San Diego, California, USA) in addition to SPSS.

Kolmogorow–Smirnow–Lilliefors test was carried out to test variables for normal distribution. Normally distributed metric data was expressed as mean ± standard deviation (SD) and analyzed using an unpaired student’s t-test. Not-normally distributed metric data was expressed as median and interquartile range (IQR); Mann–Whitney-U-test was applied for statistical analysis here. Frequencies/percentages were used for categorial data and compared using the chi-square test.

Area under the receiver operator characteristics (AUROC) curves with area under the curve (AUC) and separate analysis of Youden Index (YI) were performed using different sPAP values (sPAP 40–45–50 mmHg) and TRVmax values (TRVmax ≥ 2.9 m/s and ≥ 3.4 m/s) to determine the respective cut-off for the PA/AA-ratio.

For the analysis of inter- and intra-observer variability, the Pearson correlation coefficient with 95% confidence interval (CI) was reported for two independent investigators (radiologist 1 and 2, inter-observer variability) or two measurements taken 4 weeks apart by one investigator (radiologist 2; intra-observer variability).

Correlation analyses were absolved using Pearson correlation coefficient (metric data) or Spearman’s rank-correlation coefficient (nominal/ordinal data) to determine the strength between PA/AA or PA/AA ≥ 0.80 to further variables (age, gender, height, weight etc.).

To detect possible influencing factors regarding the presence of a potential PH with a PA/AA-ratio ≥ 0.80, a univariate, binary logistic regression analysis was figured out. For better comparability, a z-transformation was absolved for metric data. Multivariate, binary logistic regression was performed to assess independent factors regarding the prediction of a PA/AA-ratio ≥ 0.80. Therefore, covariates with *p* < 0.100 in the univariate analysis were entered and a backward variable elimination was carried out.

A Kaplan–Meier curve with a corresponding log-rank test was generated to determine whether there were differences in 1-year survival between patients with different sPAP values (≥ 40 mmHg vs. < 40 mmHg; ≥ 45 vs. < 45 mmHg; ≥ 50 mmHg vs. < 50 mmHg), TRVmax values (≥ 2.9 m/s vs. < 2.9 m/s; ≥ 3.4 m/s vs. < 3.4 m/s) and a PA/AA-ratio ≥ 0.80 compared with PA/AA-ratio < 0.80.

Univariate Cox proportional hazard regression model was used to calculate hazard ratio (HR) and 95% CI for several influencing factors associated with 1-year-mortality. Again, the z-transform was applied for metric data. Afterwards, multivariate Cox regression was performed to assess independent predictors of mortality. Therefore, again, covariates associated with mortality in the univariate analysis (*p* < 0.100) were entered and a backward variable elimination was performed.

After establishing a generalized cut-off value for the detection of a PA/AA-ratio ≥ 0.80, different expressions of biomarker plasma levels were statistically compared based on the two groups (“No PH”: PA/AA-ratio < 0.80 and “PH”: PA/AA-ratio ≥ 0.80).

Subsequently, AUROC analyses were carried out to determine an optimal cut-off value of examined cardiovascular biomarkers according to a prediction of PA/AA-ratio ≥ 0.80.

In order to investigate not only the effect of a singular biomarker, biomarkers were examined in combinations of two or three. For this purpose, a binary logistic regression was completed and the obtained values were again submitted to an AUROC analysis.

A p-value < 0.050 was considered statistically significant.

## Results

### AUROC results: sPAP/TRVmax and PA/AA-ratio

**Fig. 2:**
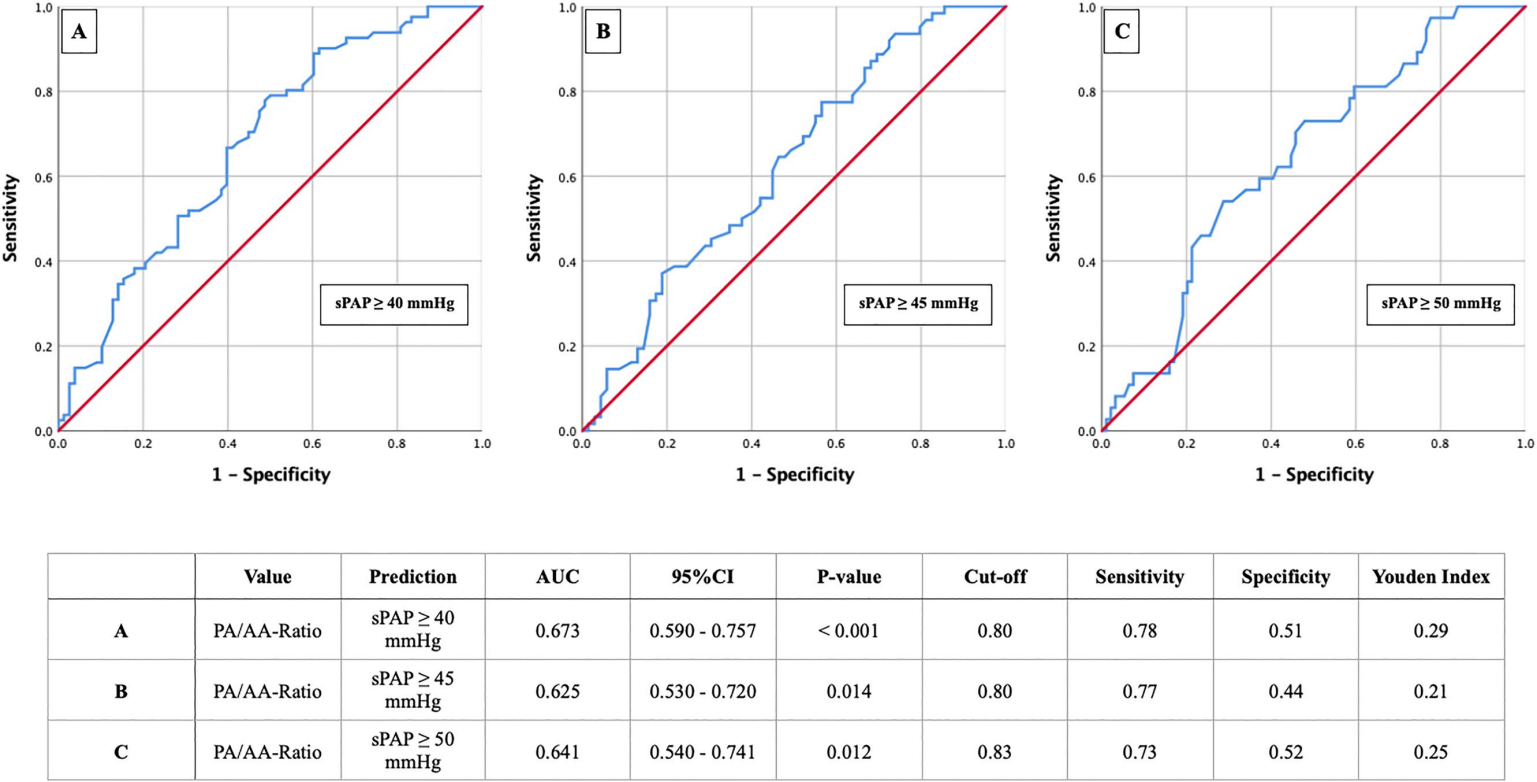
AUROC analyses of PA/AA-ratio for prediction of sPAP ≥ 40, 45 and 50 mmHg with concerning cut-off values, Youden Index, sensitivity and specificity

**Fig. 3:**
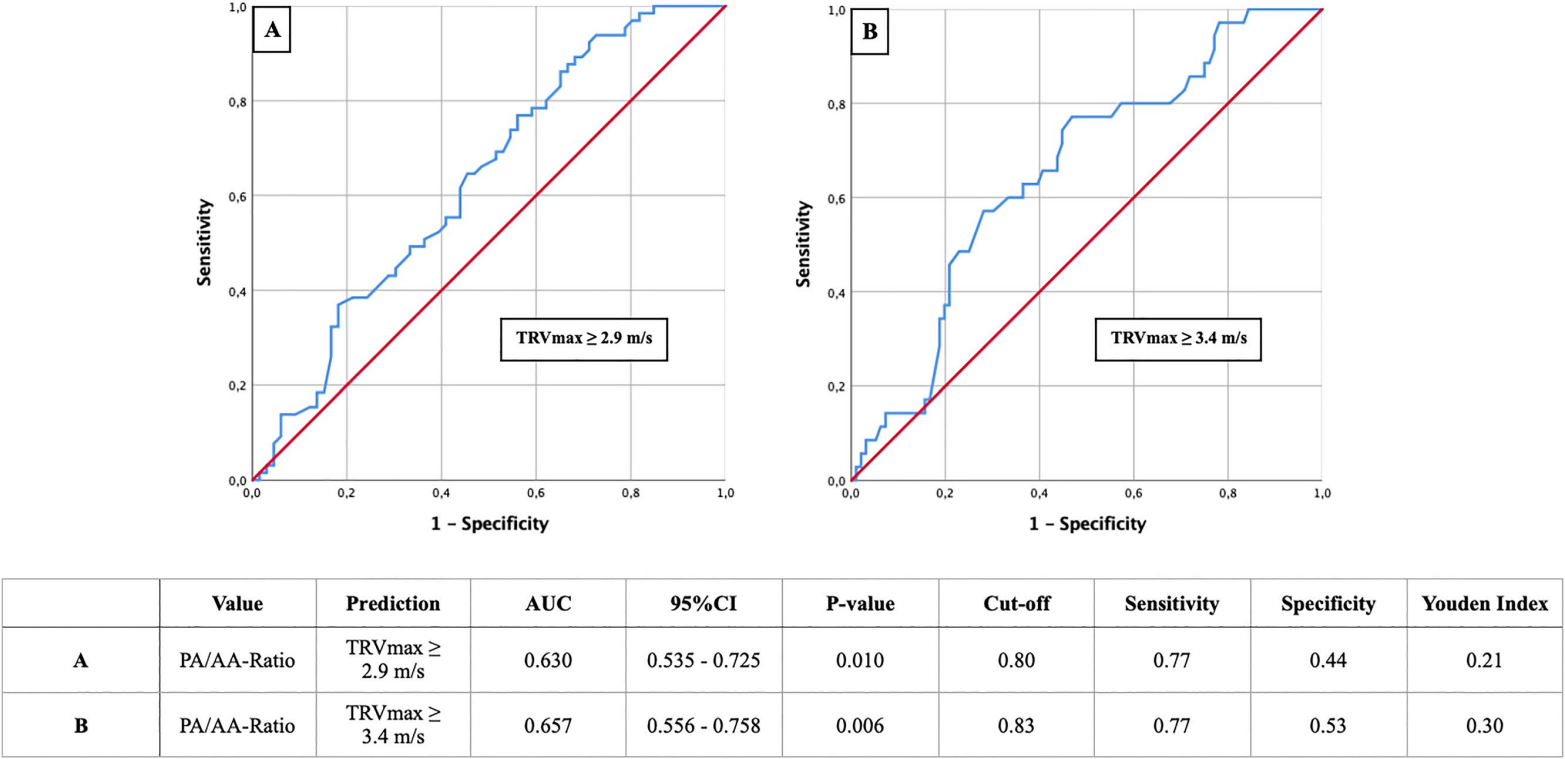
AUROC analyses of PA/AA-ratio for prediction of TRVmax ≥ 2.9 and ≥ 3.4 m/s with concerning cut-off values, Youden Index, sensitivity and specificity

In Figs. 2 and 3 the results of the primary AUROC analysis with the aim to determine the corresponding cut-offs for the PA/AA-ratio based on different sPAP values are shown.sPAP analysis identified a PA/AA-ratio of 0.80 (Fig. 2a) as an optimal cut-off value concerning an sPAP ≥ 40 mmHg (AUC 0.673; 95% CI 0.590–0.757; p < 0.001; YI 0.29; sensitivity 0.78; specificity 0.51). The sPAP values ≥ 45 mmHg and ≥ 50 mmHg provided similar PA/AA-ratio cut-off values of 0.80 and 0.83, with lower AUC and YI, respectively (Fig. 2b, c).

TRVmax analysis demonstrated similar results with a PA/AA-ratio of 0.80 (Fig. 3a) or 0.83 (Fig. 3b) when TRVmax ≥ 2.9 m/s (AUC 0.630; 95% CI 0.535–0.725; p = 0.010; YI 0.21; sensitivity 0.77; specificity 0.44) or ≥ 3.4 m/s (AUC 0.657; 95% CI 0.556–0.758; p < 0.006; YI 0.30; sensitivity 0.77; specificity 0.53) regarding corresponding guidelines were consulted.

Therefore, a value of 0.80 was used for the PA/AA-ratio in the following.

### Study cohort

194 patients with primary, severe AS from the Paracelsus Medical University Hospital Salzburg and Kepler University Hospital Linz were included in the study. 72 patients (37.11%) had a PA/AA-ratio < 0.80 in the performed ECG triggered CTA, which corresponded to the absence of PH in this study. In contrast, 122 patients (62.89%) had a PA/AA-ratio ≥ 0.80 and were listed as subjects with potential PH.

**Fig. 4:**
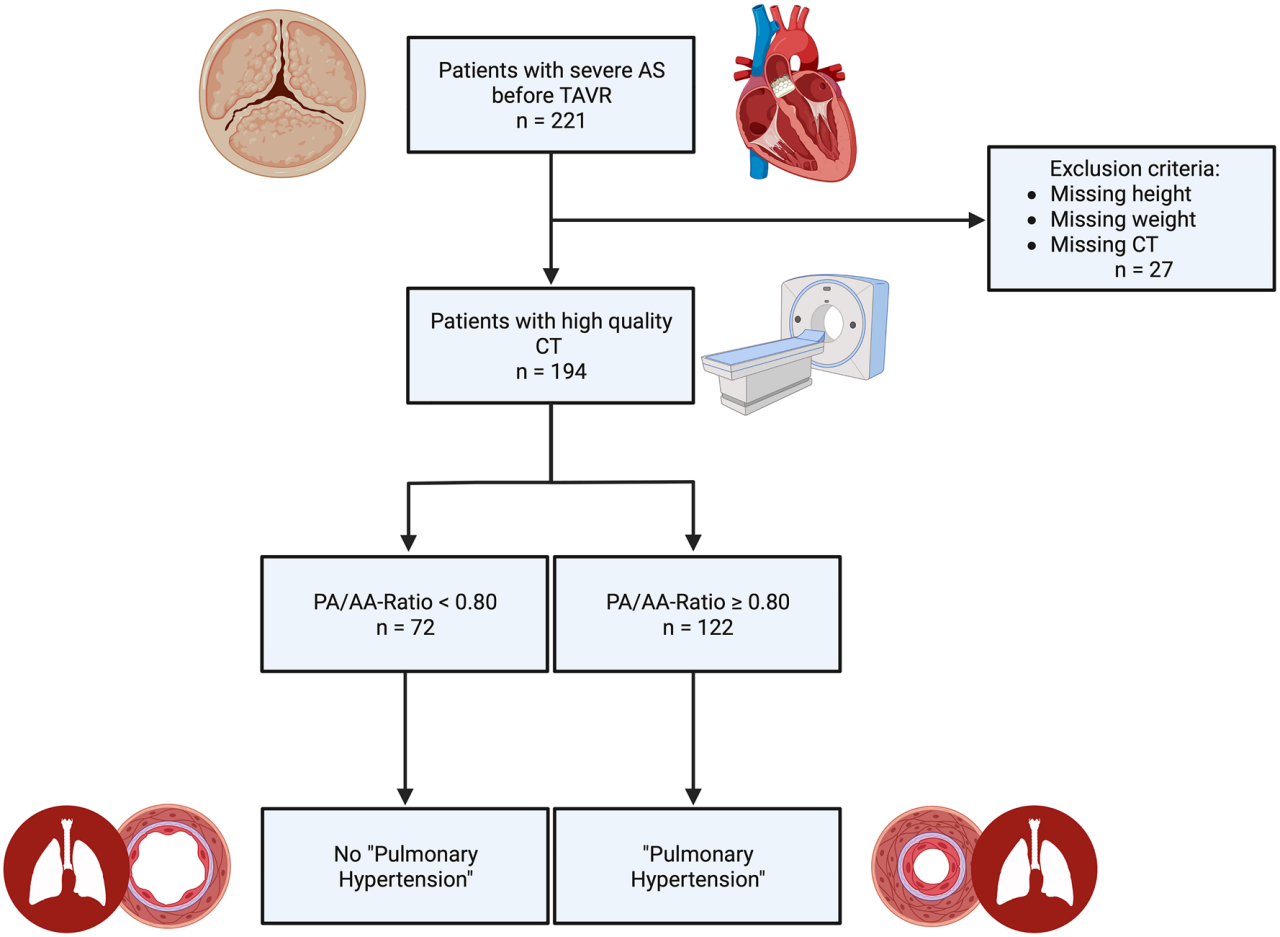
Flow chart of study inclusion and exclusion criteria (created with BioRender.com)

A flow chart regarding study inclusion and relevant exclusion criteria is shown in Fig. 4.

### Baseline characteristics

Table 1 demonstrates the collected baseline characteristics of the overall cohort and the patients classified into 2 groups according to those with a PA/AA-ratio < 0.80 and to those with a PA/AA-ratio ≥ 0.80. 52.6% of the total cohort were male, with an overall mean age of 82.8 ± 4.9 years. Significant differences between patients with a PA/AA-ratio < 0.80 vs. ≥ 0.80 were related to gender (female gender with significantly higher probability of PA/AA-ratio ≥ 0.80; p = 0.006), height (p = 0.038) and weight (p = 0.033) as well as STSScore (p = 0.025) and sPAP measurements (p = 0.018) with otherwise mostly counterbalanced further characteristics.

**Table 1:** Baseline characteristics of study population

	Overall cohort n = 194		PA/AA-ratio < 0.80 n = 72		PA/AA-ratio ≥ 0.80 n = 122		p-value
Clinical data							
Age (years)—mean ± SD	82.8	4.9	82.5	4.8	83.0	4.9	0.504
Gender (male)—%	52.6		65.3		45.1		0.006
Weight (kg)—mean ± SD	71.6	12.3	77.2	14.8	67.9	8.8	0.033
Height (cm)—mean ± SD	166.3	8.7	170.2	6.6	163.7	9.1	0.038
BMI (kg/m2)—mean ± SD	26.1	4.2	26.7	5.3	25.6	3.3	0.467
NYHA—median ± IQR	3.0	1.0	3.0	1.0	3.0	1.0	0.986
STSScore—mean ± SD	2.87	1.5	2.5	1.1	3.2	1.8	0.025
Concomitant disease							
Diabetes mellitus—%	22.2		16.7		25.4		0.157
Hypertension—%	78.9		84.7		75.5		0.125
CVD—%	72.7		79.2		68.9		0.164
CVD—1 vessel—%	22.2		25.0		20.5		0.632
CVD—2 vessels—%	9.3		9.7		9.0		0.991
CVD—3 vessels—%	9.3		11.1		8.2		0.602
Myocardial infarction—%	3.6		4.2		3.3		0.757
Atrial fibrillation—%	32.5		30.6		33.6		0.661
Pacemaker—%	7.2		8.3		6.6		0.644
Malignancy—%	21.6		22.2		21.3		0.882
Stroke—%	4.6		5.6		4.1		0.635
PAD—%	5.2		4.2		5.7		0.633
COPD—%	9.3		8.3		9.8		0.727
Echocardiography							
LVEF (%)—mean ± SD	55.7	10.8	56.8	9.8	55.1	11.4	0.303
LVEDD (mm)—mean ± SD	5.2	4.2	4.7	0.6	5.6	5.8	0.379
IVSd (mm)—mean ± SD	15.2	2.9	14.8	2.9	15.4	2.9	0.218
AV Vmax (m/s)—mean ± SD	4.4	0.6	4.4	0.5	4.3	0.6	0.660
AVdPmean (mmHg)—mean ± SD	48.7	12.4	47.9	12.7	49.2	12.3	0.498
AVdPmax (mmHg)—mean ± SD	78.0	19.1	78.1	19.5	78.0	18.9	0.960
TAPSE (mm)—mean ± SD	22.1	3.8	23.0	4.0	21.4	3.7	0.078
sPAP (mmHg)—mean ± SD	43.1	14.5	38.9	15.5	45.2	13.6	0.018
sPAP ≥ 40 mmHg—%	41.8		23.6		52.5		< 0.001
sPAP ≥ 45 mmHg—%	32.0		19.4		39.3		< 0.001
sPAP ≥ 50 mmHg—%	19.1		9.7		24.6		< 0.001
TRVmax (m/s)—mean ± SD	2.9	0.6	2.7	0.7	3.0	0.6	0.008
TRVmax ≥ 2.9 m/s—%	33.5		20.8		41.0		< 0.001
TRVmax ≥ 3.4 m/s	18.0		9.7		23.0		< 0.001
AVI ≥ II°—%	15.5		12.5		17.2		0.455
MVI ≥ II°—%	20.1		13.9		23.8		0.120
TVI ≥ II°—%	12.4		11.1		13.1		0.753
Laboratory data							
Creatinine (mg/dl)—median ± IQR	1.0	0.4	1.0	0.4	1.0	0.4	0.562
BNP (pg/ml)—median ± IQR	1174.0	2037.9	739.4	1726.2	1526.0	2416.7	0.001
cTnI (pg/ml)—median ± IQR	23.0	19.0	17.0	13.5	27.0	24.5	0.032
Hkt (%)—median ± IQR	38.7	6.1	40.1	5.7	37.8	6.9	0.013
Hb (g/dl)—median ± IQR	12.9	2.3	13.2	2.0	12.7	2.6	0.025
CK (U/l)—median ± IQR	79.0	66.0	90.5	92.3	74.0	55.0	0.014
sST2 (pg/ml)—median ± IQR	13,861.8	9114.6	13,847.7	9769.6	14,019.1	9058.8	0.934
GDF-15 (pg/ml)—median ± IQR	922.8	720.1	778.4	557.9	1025.0	736.4	0.002
H-FABP (ng/ml)—median ± IQR	1.3	1.8	1.0	1.7	1.5	1.9	0.015
IGF-BP2 (pg/ml)—median ± IQR	177,884.5	138,868.8	164,826.9	130,175.5	178,625.8	15,573.0	0.209
suPAR (pg/ml)—median ± IQR	3524.1	1861.0	3385.1	1721.2	3666.5	1850.1	0.299

*MPA* main pulmonary artery, *BMI* body mass index, *CVD* cardiovascular disease, *LVEF* left ventricular ejection fraction, *LVEDD* left ventricular end-diastolic diameter, *IVSd* interventricular septal thickness at diastole, *AV Vmax* maximal velocity over aortic valve, *AV dpmean* mean pressure gradient over aortic valve, *AV dpmax* maximal pressure gradient over aortic valve, *TAPSE* tricuspid annular plane systolic excursion, *sPAP* systolic pulmonary artery pressure, *TRVmax* maximal tricuspid regurgitation velocity, *AVI* aortic valve insufficiency, *MVI* mitral valve insufficiency, *TVI* tricuspid valve insufficiency, *BNP* brain natriuretic peptide, *cTnI* cardiac Troponin I, *Hkt* hematocrit, *Hb* hemoglobin, *CK* creatine kinase, *sST2* soluble suppression of tumorigenicity-2, *GDF-15* growth/differentiation of factor-15, *H-FABP* heart-type fatty-acid binding protein, *IGF-BP2* insulin like growth factor binding protein 2, *suPAR* soluble urokinase-type plasminogen activator receptor, *SD* standard deviation, *IQR* interquartile range

### Inter- and intra-observer variability for CT measured PA/AA-ratio

A strong and statistically highly significant (p ≤ 0.001) inter- and intra-observer correlation was found. Inter-observer variability for the pulmonary artery diameter was 0.98 (95% CI 0.96–0.98), for ascending aortic diameter 0.98 (95% CI 0.97–0.98) and for PA/AA-ratio 0.96 (95% CI 0.95–0.97). Intra-observer variability for a repeated evaluation of 50 scans for PA/AA-ratio was 0.99 (95% CI 0.99–1.00) (data not shown).

### Correlation

To investigate relationships between PA/AA-ratio or PA/AA-ratio ≥ 0.80 and other patients’ characteristics, correlation analysis using Pearson or Spearman correlation coefficient was performed (Table 2).

**Table 2:** Tabular overview of correlation analysis of PA/AA-ratio or PA/AA-ratio  ≥ 0.80 with regard to various clinical characteristics

Characteristics	PA/AA		PA/AA ≥ 0.80	
	r	p	r	p
Age	− 0.044	0.541	0.030	0.678
Gender	0.145	0.044	0.195	0.006
Weight	− 0.214	0.241	− 0.331	0.064
Height	− 0.212	0.252	− 0.377	0.036
BMI	− 0.102	0.586	− 0.102	0.584
NYHA	− 0.015	0.877	− 0.002	0.987
STSScore	0.128	0.263	0.193	0.088
Diabetes mellitus	0.107	0.138	0.102	0.158
Arterial Hypertension	− 0.073	0.314	− 0.110	0.126
CVD (all)	− 0.133	0.065	− 0.100	0.166
CVD—1 vessel	− 0.032	0.676	− 0.036	0.635
CVD—2 vessels	− 0.057	0.452	− 0.001	0.991
CVD—3 vessels	− 0.072	0.338	− 0.039	0.604
Myocardial infarction	− 0.027	0.711	− 0.022	0.759
Atrial fibrillation	0.044	0.545	0.031	0.663
Permanent pacemaker	− 0.059	0.416	− 0.033	0.646
Malignancy	0.021	0.769	− 0.011	0.882
Stroke	− 0.082	0.258	− 0.034	0.637
PAD	0.081	0.260	0.034	0.635
COPD	0.100	0.166	0.025	0.729
LVEF	− 0.088	0.226	− 0.055	0.449
LVEDD	0.153	0.205	− 0.032	0.793
IVSd	0.110	0.156	0.116	0.133
AV Vmax	− 0.064	0.409	− 0.027	0.724
AV dPmean	0.025	0.736	0.038	0.607
AV dPmax	− 0.038	0.613	0.012	0.876
TAPSE	− 0.259	0.022	− 0.212	0.062
AVI ≥ II°	0.094	0.228	0.058	0.458
MVI ≥ II°	0.076	0.300	0.113	0.121
TVI ≥ II°	0.053	0.471	0.023	0.754
sPAP	0.245	0.005	0.273	0.002
Creatinine	0.132	0.067	− 0.042	0.564
Hb	− 0.156	0.030	− 0.161	0.025
Hkt	− 0.060	0.406	− 0.178	0.013
BNP	0.301	< 0.001	0.261	< 0.001
cTnI	0.189	0.107	0.250	0.031
sST2	0.035	0.631	0.006	0.934
GDF-15	0.230	0.001	0.223	0.002
H-FABP	0.114	0.119	0.177	0.015
IGF-BP2	0.137	0.235	0.144	0.211
suPAR	0.069	0.346	0.076	0.300

*R* correlation coefficient, *PA* main pulmonary artery, *AA* ascending aorta, *BMI* body mass index, *CVD* cardiovascular disease, *PAD* peripheral artery disease, *COPD* chronic obstructive pulmonary disease, *LVEF* left ventricular ejection fraction, *LVEDD* left ventricular end diastolic diameter, *IVSd* interventricular septum diastolic, *AV max* maximal velocity over aortic valve, *AV dpmean* mean pressure gradient over aortic valve, *AV dpmax* maximal pressure gradient over aortic valve, *TAPSE AVI* aortic valve insufficiency, MVI mitral valve insufficiency, *TVI* tricuspid valve insufficiency, *sPAP* systolic pulmonary arterial pressure, *Hb* hemoglobin, *Hkt* hematocrit, *BNP* brain natriuretic peptide, *cTnI* cardiac troponin I, *sST2* soluble suppression of tumorigenicity-2, *GDF-15* growth/differentiation of factor-15, *H-FABP* heart-type fatty-acid binding protein, *IGF-BP2* insulin like growth factor binding protein 2, *suPAR* soluble urokinase-type plasminogen activator receptor

Overall, both the PA/AA ratio and the PA/AA ratio ≥ 0.80 generally showed no pronounced correlations with the investigated clinical variables. The highest positive correlation was found for BNP (PA/AA-ratio r: 0.301, p < 0.001; PA/AA-ratio ≥ 0.80 r: 0.261, p < 0.001) and sPAP (PA/AA-ratio r: 0.245, p = 0.005; PA/AA-ratio r: 0.273, p = 0.002).

### Binary logistic regression

In order to verify a relevant statistical relationship between the potential presence of PH via PA/AA-ratio ≥ 0.80 and other factors (especially gender, weight, height etc.), a univariate as well as a multivariate binary logistic regression was performed (Table 3).

**Table 3:** Univariate and multivariate, binary, logistic regression analysis detecting predictors of potential PH via PA/AA-ratio  ≥ 0.80

PA/AA ≥ 0.80 Binary logistic regression	Univariate		Multivariate	
	Hazard ratio (95% CI)	p-value	Hazard ratio (95% CI)	p-value
Age	1.021 (0.961–1.084)	0.502		
Gender (male)	0.437 (0.239–0.797)	0.007	0.678 (0.463–0.756)	0.462
Weight	0.403 (0.162–1.004)	0.051	0.947 (0.848–1.057)	0.332
Height	0.419 (0.176–0.995)	0.049	0.916 (0.796–1.054)	0.219
BMI	0.935 (0.785–1.114)	0.455		
NYHA	1.028 (0.523–2.022)	0.935		
STSScore	1.762 (1.021–3.042)	0.042	0.869 (0.319–2.371)	0.785
Diabetes mellitus	1.703 (0.811–3.576)	0.159		
Arterial Hypertension	0.553 (0.258–1.186)	0.128		
CVD (all)	0.614 (0.308–1.224)	0.166		
CVD—1 vessel	0.843 (0.419–1.696)	0.632		
CVD—2 vessels	0.994 (0.366–2.701)	0.991		
CVD—3 vessels	0.770 (0.288–2.058)	0.603		
Myocardial infarction	0.786 (0.171–3.618)	0.758		
Atrial fibrillation	1.150 (0.615–2.153)	0.661		
Permanent pacemaker	0.772 (0.257–2.321)	0.645		
Malignancy	0.948 (0.469–1.918)	0.882		
Stroke	0.722 (0.187–2.781)	0.636		
PAD	1.400 (0.350–5.593)	0.634		
COPD	1.200 (0.430–3.349)	0.728		
LVEF	0.985 (0.958–1.013)	0.302		
LVEDD	1.100 (0.812–1.491)	0.538		
IVSd	1.071 (0.960–1.194)	0.218		
AV Vmax	0.882 (0.506–1.538)	0.658		
AV dpmean	1.008 (0.984–1.033)	0.496		
AV dpmax	1.000 (0.984–1.015)	0.960		
TAPSE	0.897 (0.794–1.013)	0.181		
AVI ≥ II°	1.384 (0.589–3.251)	0.456		
MVI ≥ II°	1.860 (0.844–4.096)	0.124		
TVI ≥ II°	1.157 (0.467–2.866)	0.753		
sPAP	1.663 (1.077–2.568)	0.022	1.015 (0.924–1.114)	0.760
Creatinine	0.896 (0.358–2.241)	0.814		
Hb	0.685 (0.503–0.933)	0.016	39.578 (0.363–4309.860)	0.124
Hkt	0.098 (0.015–0.659)	0.017	0.000 (0.000–235.088)	0.123
BNP	1.833 (1.100–3.053)	0.020	0.931 (0.196–4.428)	0.929
cTnI	2.438 (1.109–5.359)	0.027	3.392 (0.700–16.439)	0.129
sST2	0.995 (0.741–1.336)	0.975		
GDF-15	1.662 (1.153–2.395)	0.006	0.960 (0.120–7.696)	0.969
H-FABP	1.278 (0.892–1.832)	0.181		
IGF-BP2	1.387 (0.757–2.540)	0.290		
suPAR	1.086 (0.803–1.469)	0.594		

*PA* main pulmonary artery, *AA* ascending aorta, *BMI* body mass index, *CVD* cardiovascular disease, *PAD* peripheral artery disease, *COPD* chronic obstructive pulmonary disease, *LVEF* left ventricular ejection fraction, *LVEDD* left ventricular end diastolic diameter, *IVSd* interventricular septum diastolic, *AV max* maximal velocity over aortic valve, *AV dpmean* mean pressure gradient over aortic valve, *AV dpmax* maximal pressure gradient over aortic valve, *TAPSE AVI* aortic valve insufficiency, *MVI* mitral valve insufficiency, *TVI* tricuspid valve insufficiency, *sPAP* systolic pulmonary arterial pressure, *Hb* hemoglobin, *Hkt* hematocrit, *BNP* brain natriuretic peptide, *cTnI* cardiac troponin I, *sST2* soluble suppression of tumorigenicity-2, *GDF-15* growth/differentiation of factor-15, *H-FABP* heart-type fatty-acid binding protein, *IGF-BP2* insulin like growth factor binding protein 2, *suPAR* soluble urokinase-type plasminogen activator receptor

In the univariate analysis, gender, weight, height, STSScore, sPAP, hemoglobin, hematocrit, BNP, cTnI and GDF-15 showed a relevant association (*p* < 0.100), so multivariate analysis was performed with these variables. None of the clinical characteristics finally showed a *p* value ≤ 0.050.

### Kaplan–Meier results

**Fig. 5:**
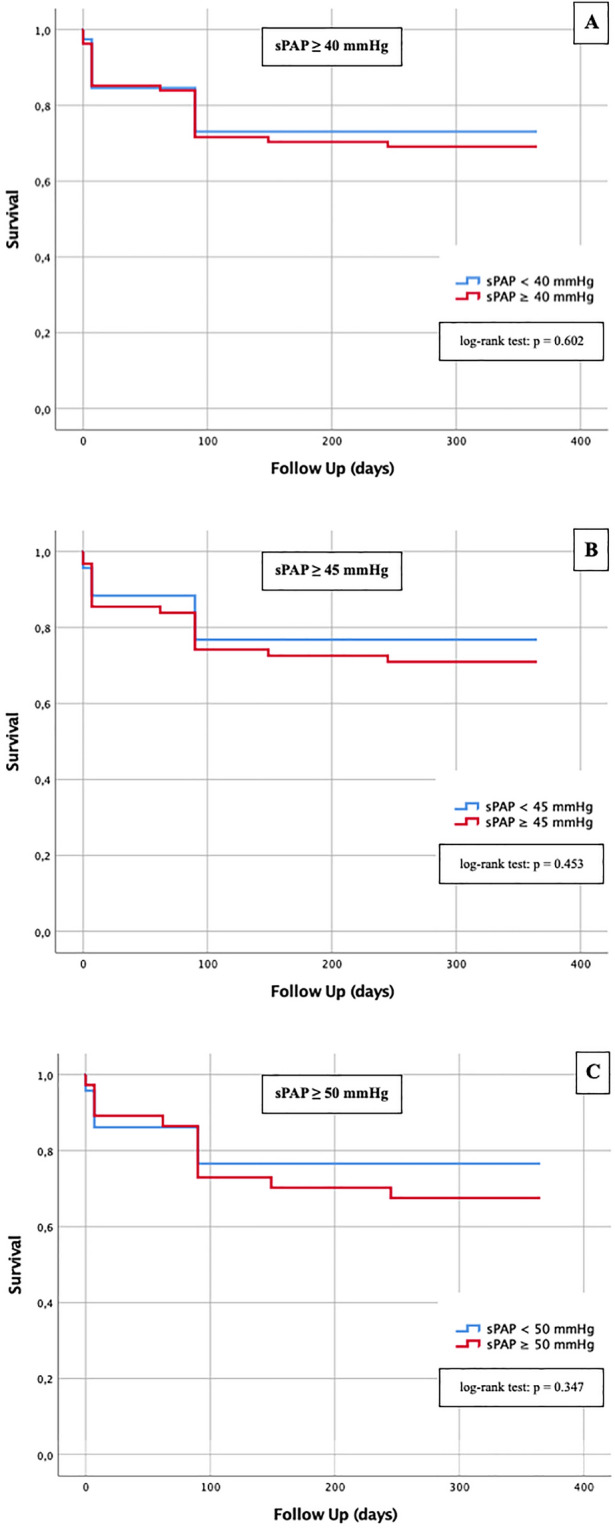
Kaplan–Meier curves for detection of 1-year survival in dependence of different sPAP cut-off values; **a** sPAP ≥ 40 mmHg; **b** sPAP ≥ 45 mmHg; **c** sPAP ≥ 50 mmHg

**Fig. 6:**
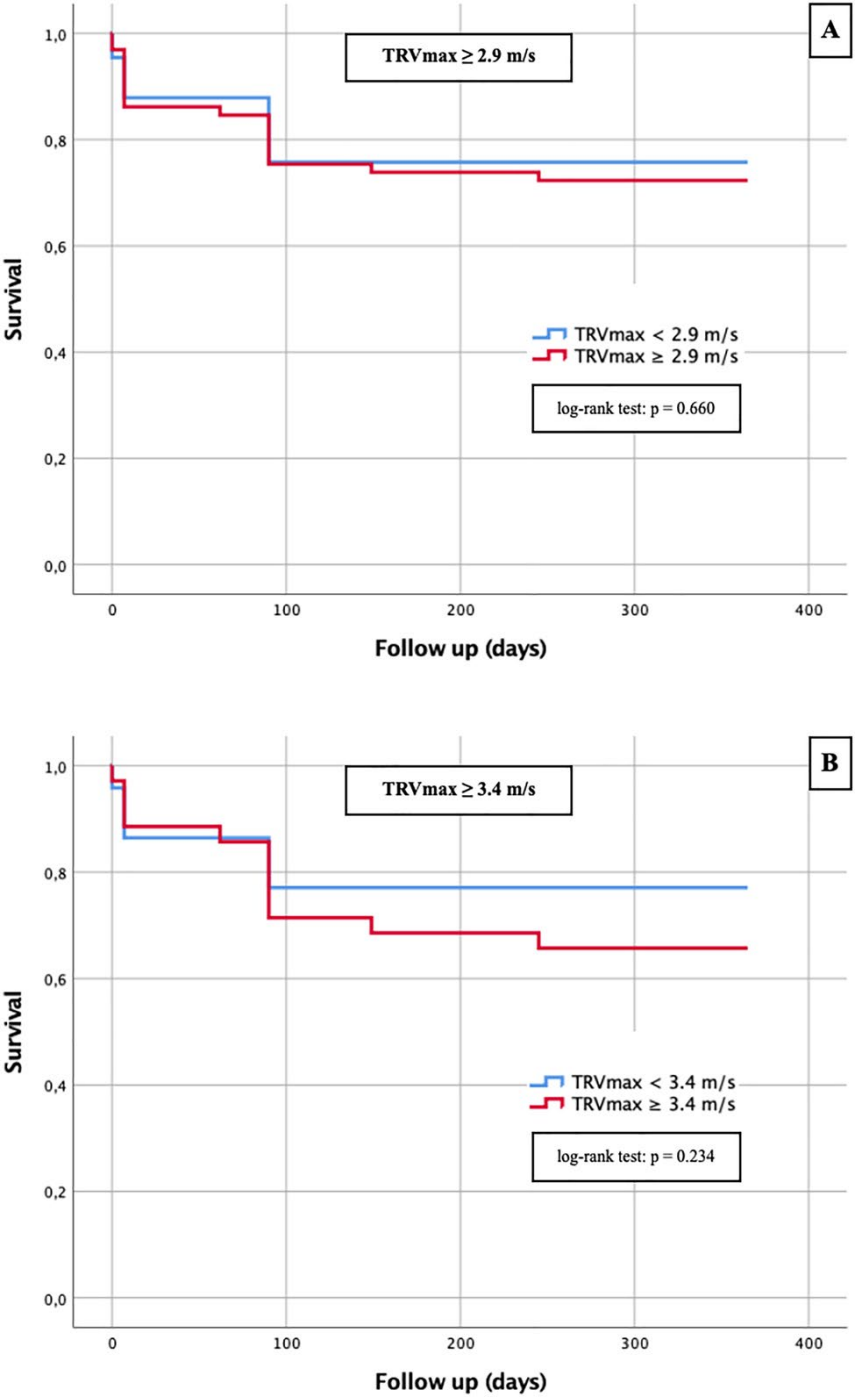
Kaplan–Meier curves for detection of 1-year survival in dependence of different TRVmax cut-off values; **a** TRVmax ≥ 2.9 m/s; **b** TRVmax ≥ 3.4 m/s

**Fig. 7:**
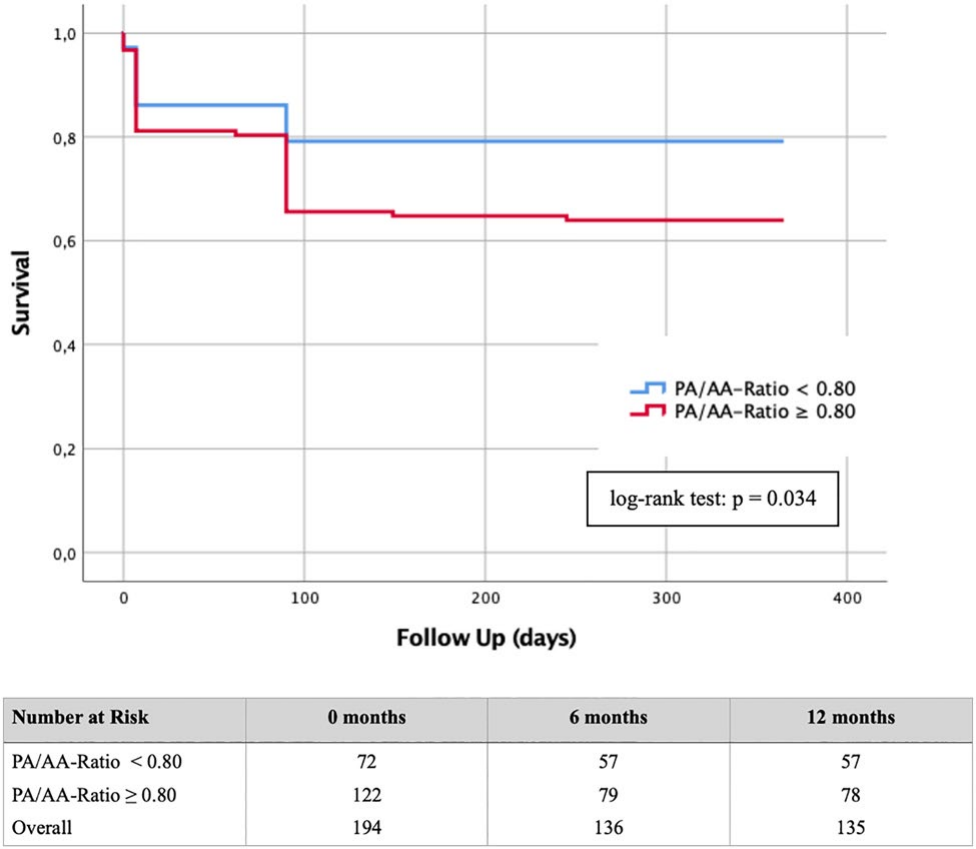
Kaplan–Meier curve with corresponding numbers at risk for detection of 1-year survival in dependence of a PA/AA-ratio cut-off value ≥ 0.80

Kaplan Meier curves were performed with special attention regarding 1-year survival depending on different sPAP values (Fig. 5), TRVmax values (Fig. 6) and the PA/AA-ratio determined by AUROC (Fig. 7).

The sPAP was not a relevant predictor with respect to 1-year survival regardless of the threshold chosen, as the log-rank test for sPAP ≥ 40 mmHg was p = 0.602 (Fig. 5a), for sPAP ≥ 45 mmHg p = 0.453 (Fig. 5b) and for sPAP ≥ 50 mmHg p = 0.347 and thus each not statistically significant. Similarly, the Kaplan–Meier curves for TRVmax ≥ 2.9 m/s (Fig. 6a; log-rank p = 0.660) and TRVmax ≥ 3.4 m/s (Fig. 6b; log-rank p = 0.234) were not statistically significant.

Patients with a PA/AA-ratio < 0.80 showed a significant lower mortality during the first year after TAVR then the corresponding group with a PA/AA-ratio ≥ 0.80 (log-rank test: p = 0.034). In the group of patients with a PA/AA-ratio < 0.80, 15/72 (20.83%) died within one year. In contrast, 44/122 (36.07%) from the group with a PA/AA-ratio ≥ 0.80 passed away.

### Cox hazard regression

To investigate influencing factors concerning 1-year mortality after TAVR, a univariate and multivariate Cox proportional hazard regression was figured out (Table 4). The result of univariate analyses showed agreement (*p* < 0.100) with atrial fibrillation, mitral valve insufficiency and PA/AA-ratio ≥ 0.80. After inclusion of these data in a multivariate analysis, atrial fibrillation (*p* = 0.045) and PA/AA-ratio ≥ 0.80 (*p* = 0.042) remained independent factors for estimation of mortality after 1 year.

**Table 4:** Univariate and multivariate Cox hazard regression analysis detecting predictors of 1-year mortality

1-Year mortality Cox regression analysis	Univariate		Multivariate	
	Hazard ratio (95% CI)	p-value	Hazard ratio (95% CI)	p-value
Age	1.026 (0.972–1.083)	0.356		
Gender (male)	0.926 (0.555–1.545)	0.767		
Weight	1.039 (0.975–1.108)	0.240		
Height	1.044 (0.927–1.175)	0.483		
BMI	1.082 (0.883–1.326)	0.448		
NYHA	0.722 (0.431–1.208)	0.214		
STSScore	0.742 (0.403–1.368)	0.339		
Diabetes mellitus	1.008 (0.545–1.865)	0.980		
Arterial Hypertension	1.196 (0.621–2.303)	0.592		
CVD (all)	1.493 (0.792–2.814)	0.215		
CVD—1 vessel	1.381 (0.768–2.483)	0.280		
CVD—2 vessels	0.516 (0.161–1.654)	0.266		
CVD—3 vessels	0.310 (0.076–1.275)	0.105		
Myocardial infarction	0.968 (0.236–3.967)	0.964		
Atrial fibrillation	1.731 (1.035–2.896)	0.037	1.692 (1.011–2.832)	0.045
Permanent pacemaker	1.297 (0.519–3.243)	0.578		
Malignancy	1.1013 (0.547–1.875)	0.967		
Stroke	1.621 (0.587–4.475)	0.351		
PAD	0.641 (0.156–2.625)	0.536		
COPD	1.559 (0.740–3.285)	0.243		
LVEF	0.994 (0.971–1.018)	0.624		
LVEDD	0.892 (0.317–2.505)	0.828		
IVSd	1.185 (1.083–1.296)	0.111		
AV Vmax	1.337 (0.803–2.226)	0.264		
AV dpmean	1.012 (0.992–1.032)	0.237		
AV dpmax	1.004 (0.990–1.017)	0.591		
TAPSE	0.926 (0.797–1.076)	0.318		
AVI ≥ II°	1.623 (0.734–3.588)	0.231		
MVI ≥ II°	2.070 (0.940–4.557)	0.071	2.189 (0.991–4.834)	0.053
TVI ≥ II°	1.298 (0.557–3.025)	0.546		
sPAP	1.008 (0.987–1.030)	0.469		
PA/AA ≥ 0.80	1.797 (1.000–3.230)	0.050	1.840 (1.022–3.313)	0.042
PA/AA	2.384 (0.416–13.670)	0.330		
PA	1.027 (0.979–1.076)	0.272		
AA	1.000 (0.942–1.062)	0.999		
Creatinine	1.052 (0.471–2.351)	0.901		
Hb	0.982 (0.760–1.269)	0.889		
Hkt	0.800 (0.320–2.000)	0.633		
BNP	1.060 (0.840–1.338)	0.624		
cTnI	0.883 (0.403–1.936)	0.756		
sST2	1.181 (0.927–1.504)	0.177		
GDF-15	1.143 (0.897–1.456)	0.280		
H-FABP	1.080 (0.868–1.343)	0.491		
IGF-BP2	1.448 (0.974–2.153)	0.107		
suPAR	0.913 (0.690–1.210)	0.528		

*BMI* body mass index, *CVD* cardiovascular disease, *PAD* peripheral artery disease, *COPD* chronic obstructive pulmonary disease, *LVEF* left ventricular ejection fraction, *LVEDD* left ventricular end diastolic diameter, *IVSd* interventricular septum diastolic, *AV max* maximal velocity over aortic valve, *AV dpmean* mean pressure gradient over aortic valve, *AV dpmax* maximal pressure gradient over aortic valve, *TAPSE AVI* aortic valve insufficiency, *MVI* mitral valve insufficiency, *TVI* tricuspid valve insufficiency, *sPAP* systolic pulmonary arterial pressure, *PA* main pulmonary artery, *AA* ascending aorta, *Hb* hemoglobin, *Hkt* hematocrit, *BNP* brain natriuretic peptide, *cTnI* cardiac troponin I, *sST2* soluble suppression of tumorigenicity-2, *GDF-15* growth/differentiation of factor-15, *H-FABP* heart-type fatty-acid binding protein, *IGF-BP2* insulin like growth factor binding protein 2, *suPAR* soluble urokinase-type plasminogen activator receptor

### Biomarker concentrations in dependence of new PA/AA-ratio cut-off value

**Fig. 8:**
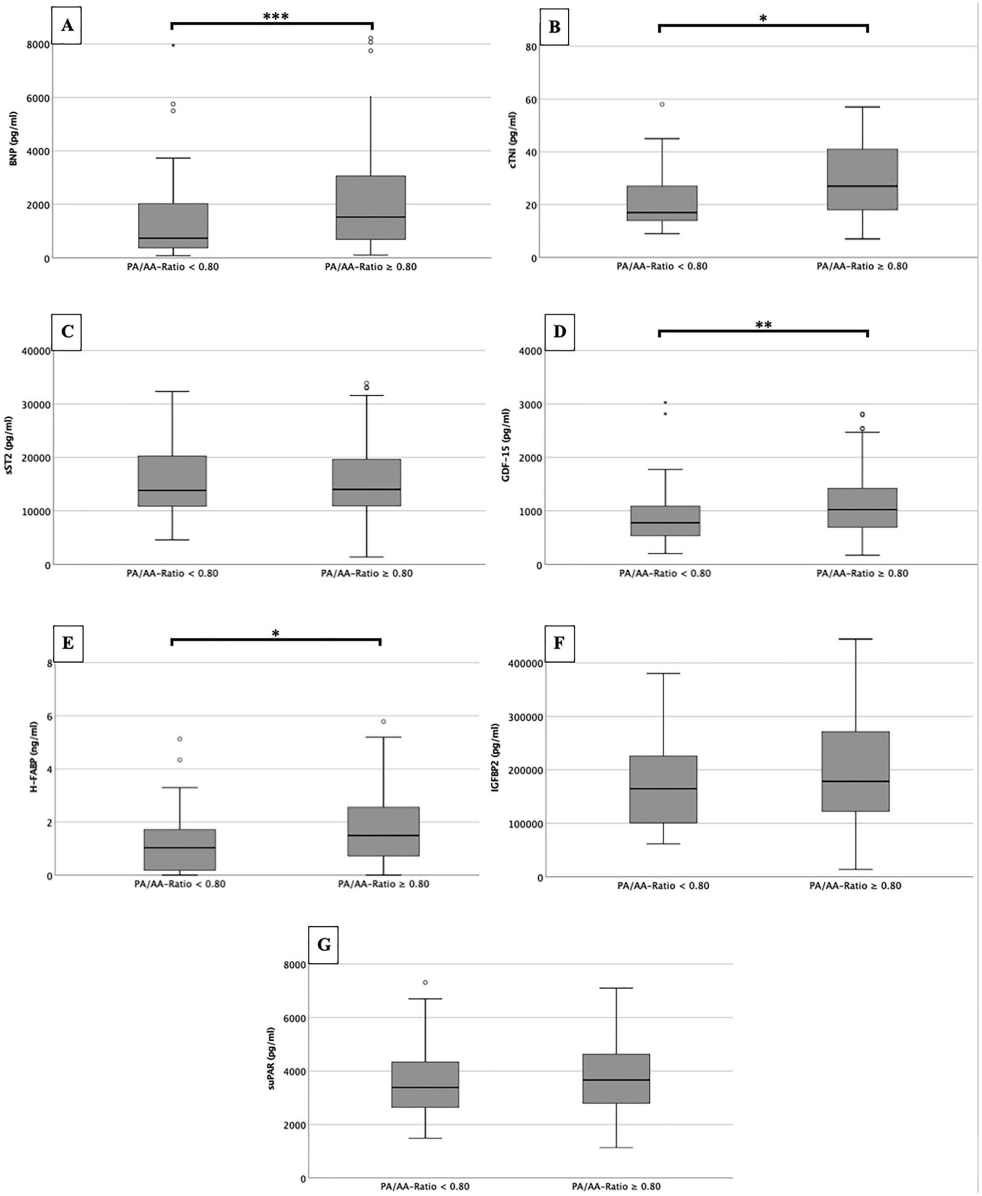
Serum concentrations of BNP, cTnI, sST2, GDF-15, H-FABP, IGF-BP2 and suPAR in patients with a PA/AA-ratio  ≥ 0.80 and with a PA/AA_ratio_ ≥ 0.80; *p ≤ 0.05; **p ≤ 0.01; ***≤ 0.001

Figure 8 summarizes the corresponding plasma concentrations of the determined cardiovascular biomarkers depending on the PA/AA-ratio obtained (≥ 0.80 vs. < 0.80).

BNP (Fig. 8a) demonstrated significantly higher plasma concentrations in patients with a PA/AA-ratio  ≥ 0.80 than with a PA/AA-ratio  < 0.80 (1526.0 ± 2416.7 pg/ml vs. 739.4 ± 1726.2 pg/ml; p = 0.001). Equally significant results could be found for cTnI (Fig. 8b) with plasma concentrations of 27.0 ± 24.5 pg/ml vs. 17.0 ± 13.5 pg/ml (p = 0.032), for GDF-15 (Fig. 8d) with 1025.0 ± 736.4 pg/ml vs. 778.4 ± 557.9 pg/ml (p = 0.002) and for H-FABP with 1.5 ± 1.9 ng/ml vs. 1.0 ± 1.7 ng/ml (Fig. 8e).

Other biomarkers studied, such as sST2 (Fig. 6c), IGF-BP2 (Fig. 8f) and suPAR (Fig. 8g) did not show significant differences between the two different PA/AA-ratio groups.

### AUROC results: PA/AA-ratio and singular cardiovascular biomarker

**Fig. 9:**
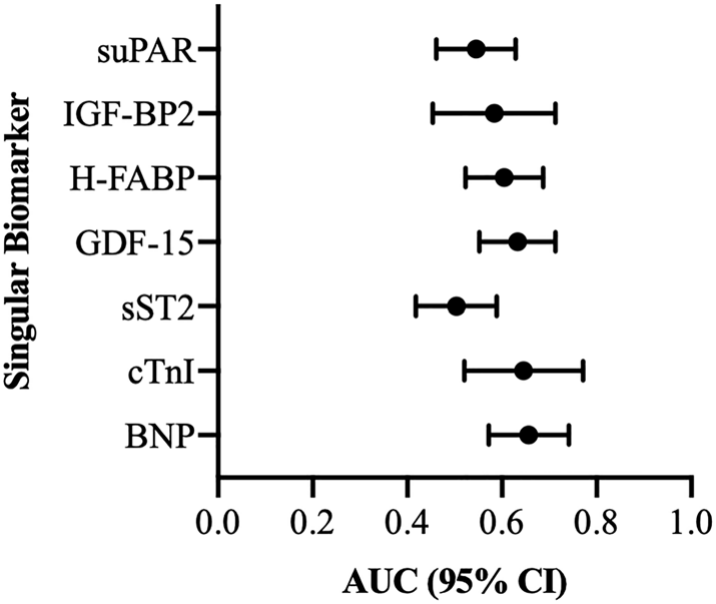
AUROC analyses of BNP, cTnI GDF-15, H-FABP, IGF-BP2 and suPAR for prediction of a PA/AA-ratio  ≥ 0.80

To analyze potential biomarkers for prediction of a PA/AA-ratio  ≥ 0.80 in patients with severe AS before TAVR, AUROC-curves regarding plasma level concentration of BNP, cTnI, sST2, GDF-15, H-FABP, IGF-BP2 and suPAR were figured out. Therefore AUC, cut-off values with YI as well as sensitivity and specificity were extracted (Fig. 9; Table 5).

**Table 5:** AUROC analyses of singular biomarkers for prediction of a PA/AA-ratio  ≥ 0.80 with concerning Youden Index, sensitivity and specificity

Value	Prediction	AUC	95% CI	p-value	Cut-off	Sensitivity	Specificity	Youden Index
suPAR (pg/ml)	PA/AA-ratio ≥ 0.80	0.545	0.461–0.629	0.299	3917.41	0.45	0.70	0.15
IGF-BP2 (pg/ml)	PA/AA-ratio ≥ 0.80	0.584	0.454–0.713	0.209	106,416.89	0.84	0.38	0.22
H-FABP (ng/ml)	PA/AA-ratio ≥ 0.80	0.605	0.523–0.687	0.016	1.05	0.67	0.55	0.22
GDF-15 (pg/ml)	PA/AA-ratio ≥ 0.80	0.633	0.552–0.713	0.002	1118.41	0.44	0.79	0.23
sST2 (pg/ml)	PA/AA-ratio ≥ 0.80	0.504	0.418–0.589	0.504	11,686.06	0.69	0.38	0.07
cTnI (pg/ml)	PA/AA-ratio ≥ 0.80	0.645	0.520–0.771	0.033	17.50	0.76	0.52	0.27
BNP (pg/ml)	PA/AA-ratio ≥ 0.80	0.656	0.572–0.741	0.001	625.30	0.82	0.46	0.28

BNP: brain natriuretic peptide; cTnI: cardiac Troponin I; sST2: soluble suppression of tumorigenicity-2; GDF-15: growth/differentiation of factor-15; H-FABP: heart-type fatty-acid binding protein; IGF-BP2: insulin like growth factor binding protein 2; suPAR: soluble urokinase-type plasminogen activator receptor

AUROC analyses showed significant values for BNP, cTnI, GDF-15 and H-FABP showing the best AUC on the side of BNP (Fig. 9—AUC 0.656; 95% CI 0.572–0.741; YI 0.28; sensitivity 0.82; specificity 0.46; p = 0.001). sST2, IGF-BP2 and suPAR were not statistically significant with respect to their determined AUROC results.

### AUROC results: PA/AA-ratio and multiple combinations of cardiovascular biomarkers

**Fig. 10:**
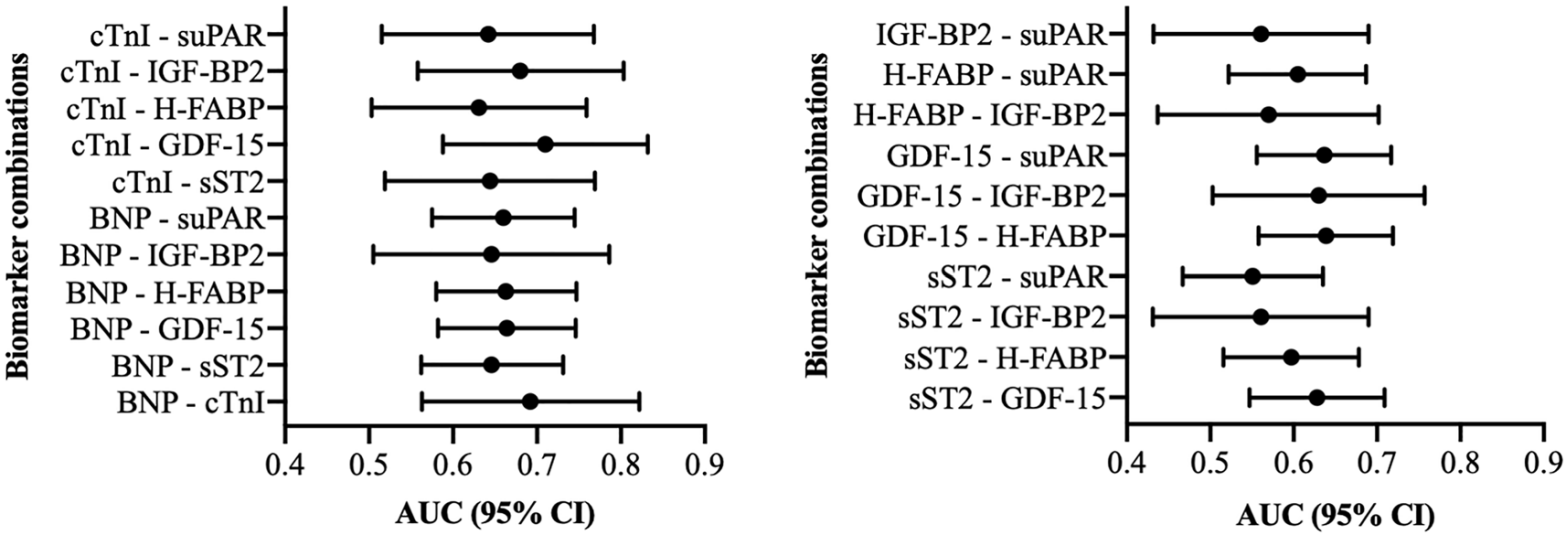
Two-way biomarker combinations with corresponding AUROC analyses

**Fig. 11:**
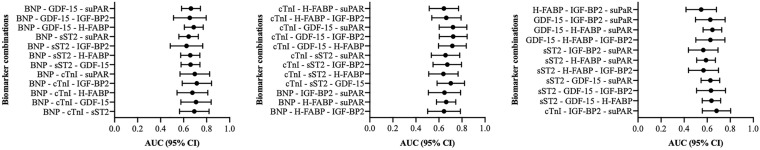
Three-way biomarker combinations with corresponding AUROC analyses

For a better overview and also considering clinical practicability, 2 (Fig. 10; Table 6) or 3 (Fig. 11; Table 7) biomarkers were examined in combination and AUROC analyses were performed.

**Table 6:** AUROC analyses of a combination of 2 biomarkers for prediction of a PA/AA-ratio  ≥ 0.80 with concerning Youden Index, sensitivity and specificity

Values	Prediction	AUC	95% CI	p-value	Sensitivity	Specificity	Youden Index
IGF-BP2 + suPAR	PA/AA-ratio ≥ 0.80	0.561	0.432–0.690	0.361	0.72	0.44	0.16
H-FABP + suPAR	PA/AA-ratio ≥ 0.80	0.605	0.522–0.687	0.016	0.59	0.62	0.21
H-FABP + IGF-BP2	PA/AA-ratio ≥ 0.80	0.570	0.437–0.702	0.302	0.76	0.44	0.20
GDF-15 + suPAR	PA/AA-ratio ≥ 0.80	0.637	0.556–0.717	0.002	0.71	0.58	0.29
GDF-15 + IGF-BP2	PA/AA-ratio ≥ 0.80	0.630	0.503–0.757	0.054	0.56	0.77	0.33
GDF-15 + H-FABP	PA/AA-ratio ≥ 0.80	0.639	0.558–0.719	0.001	0.51	0.76	0.27
sST2 + suPAR	PA/AA-ratio ≥ 0.80	0.551	0.467–0.635	0.243	0.53	0.70	0.23
sST2 + IGF-BP2	PA/AA-ratio ≥ 0.80	0.561	0.431–0.690	0.361	0.51	0.65	0.16
sST2 + H-FABP	PA/AA-ratio ≥ 0.80	0.597	0.516–0.678	0.026	0.40	0.82	0.22
sST2 + GDF-15	PA/AA-ratio ≥ 0.80	0.628	0.547–0.709	0.003	0.63	0.63	0.27
cTnI + suPAR	PA/AA-ratio ≥ 0.80	0.642	0.515–0.768	0.037	0.66	0.67	0.33
cTnI + IGF-BP2	PA/AA-ratio ≥ 0.80	0.680	0.558–0.803	0.009	0.60	0.75	0.35
cTnI + H-FABP	PA/AA-ratio ≥ 0.80	0.631	0.503–0.759	0.057	0.74	0.52	0.26
cTnI + GDF-15	PA/AA-ratio ≥ 0.80	0.710	0.588–0.832	0.002	0.69	0.73	0.42
cTnI + ssT2	PA/AA-ratio ≥ 0.80	0.644	0.519–0.769	0.034	0.46	0.82	0.28
BNP + suPAR	PA/AA-ratio ≥ 0.80	0.660	0.575–0.745	< 0.001	0.77	0.53	0.30
BNP + IGF-BP2	PA/AA-ratio ≥ 0.80	0.646	0.505–0.786	0.051	0.57	0.78	0.35
BNP + H-FABP	PA/AA-ratio ≥ 0.80	0.663	0.580–0.747	< 0.001	0.73	0.56	0.30
BNP + GDF-15	PA/AA-ratio ≥ 0.80	0.664	0.582–0.746	< 0.001	0.44	0.86	0.30
BNP + sST2	PA/AA-ratio ≥ 0.80	0.646	0.562–0.731	0.001	0.57	0.72	0.29
BNP + cTnI	PA/AA-ratio ≥ 0.80	0.692	0.563–0.822	0.009	0.56	0.79	0.34

*BNP* brain natriuretic peptide, *cTnI* cardiac Troponin I, *sST2* soluble suppression of tumorigenicity-2, *GDF-15* growth/differentiation of factor-15, *H-FABP* heart-type fatty-acid binding protein, *IGF-BP2* insulin like growth factor binding protein 2, *suPAR* soluble urokinase-type plasminogen activator receptor

**Table 7:** AUROC analyses of a combination of 3 biomarkers for prediction of a PA/AA-ratio  ≥ 0.80 with concerning Youden Index, sensitivity and specificity

Values	Prediction	AUC	95% CI	p-value	Sensitivity	Specificity	Youden Index
H-FABP—IGF-BP2—suPAR	PA/AA-ratio ≥ 0.80	0.548	0.416–0.680	0.467	0.66	0.53	0.19
GDF-15—IGF-BP2—suPAR	PA/AA-ratio ≥ 0.80	0.627	0.499–0.755	0.060	0.54	0.77	0.31
GDF-15—H-FABP—suPAR	PA/AA-ratio ≥ 0.80	0.645	0.565–0.726	0.001	0.68	0.61	0.28
GDF-15—H-FABP—IGF-BP2	PA/AA-ratio ≥ 0.80	0.628	0.501–0.754	0.058	0.39	0.88	0.27
sST2—IGF-BP2—suPAR	PA/AA-ratio ≥ 0.80	0.566	0.437–0.694	0.065	0.37	0.79	0.17
sST2- H-FABP—suPAR	PA/AA-ratio ≥ 0.80	0.590	0.509–0.671	0.039	0.60	0.60	0.20
sST2—H-FABP—IGF-BP2	PA/AA-ratio ≥ 0.80	0.568	0.438–0.698	0.312	0.54	0.65	0.19
sST2—GDF-15—suPAR	PA/AA-ratio ≥ 0.80	0.628	0.547–0.709	0.003	0.66	0.62	0.28
sST2—GDF-15—IGF-BP2	PA/AA-ratio ≥ 0.80	0.633	0.508–0.759	0.048	0.37	0.91	0.28
sST2—GDF-15—H-FABP	PA/AA-ratio ≥ 0.80	0.637	0.557–0.717	0.002	0.35	0.89	0.24
cTnI—IGF-BP2—suPAR	PA/AA-ratio ≥ 0.80	0.681	0.558–0.804	0.009	0.63	0.72	0.34
cTnI—H-FABP—suPAR	PA/AA-ratio ≥ 0.80	0.643	0.515–0.770	0.038	0.82	0.45	0.28
cTnI—H-FABP—IGF-BP2	PA/AA-ratio ≥ 0.80	0.664	0.538–0.792	0.018	0.58	0.72	0.30
cTnI—GDF-15—suPAR	PA/AA-ratio ≥ 0.80	0.722	0.602–0.842	0.001	0.67	0.76	0.43
cTnI—GDF-15—IGF-BP2	PA/AA-ratio ≥ 0.80	0.724	0.603–0.845	0.001	0.71	0.72	0.43
cTnI—GDF-15—H-FABP	PA/AA-ratio ≥ 0.80	0.716	0.596–0.837	0.002	0.62	0.82	0.44
cTnI—sST2—suPAR	PA/AA-ratio ≥ 0.80	0.657	0.534–0.780	0.021	0.44	0.82	0.26
cTnI—sST2 -IGF-BP2	PA/AA-ratio ≥ 0.80	0.673	0.549–0.796	0.012	0.48	0.91	0.39
cTnI sST2—H-FABP	PA/AA-ratio ≥ 0.80	0.639	0.511–0.766	0.044	0.44	0.85	0.29
cTnI—sST2—GDF-15	PA/AA-ratio ≥ 0.80	0.703	0.584–0.822	0.003	0.44	0.94	0.38
BNP—IGF-BP2—suPAR	PA/AA-ratio ≥ 0.80	0.648	0.508–0.788	0.048	0.60	0.74	0.34
BNP—H-FABP—suPAR	PA/AA-ratio ≥ 0.80	0.663	0.579–0.746	< 0.001	0.73	0.56	0.29
BNP—H-FABP—IGF-BP2	PA/AA-ratio ≥ 0.80	0.644	0.502–0.786	0.056	0.76	0.52	0.28
BNP—GDF-15—suPAR	PA/AA-ratio ≥ 0.80	0.664	0.582–0.746	< 0.001	0.58	0.72	0.30
BNP—GDF-15-IGF-BP2	PA/AA-ratio ≥ 0.80	0.654	0.512–0.796	0.041	0.55	0.82	0.37
BNP—GDF-15—H-FABP	PA/AA-ratio ≥ 0.80	0.688	0.606–0.770	< 0.001	0.77	0.58	0.35
BNP—sST2—suPAR	PA/AA-ratio ≥ 0.80	0.644	0.559–0.728	0.002	0.58	0.72	0.30
BNP—sST2—IGF-BP2	PA/AA-ratio ≥ 0.80	0.625	0.484–0.766	0.092	0.63	0.70	0.33
BNP—sST2—H-FABP	PA/AA-ratio ≥ 0.80	0.658	0.575–0.741	0.001	0.53	0.73	0.26
BNP—sST2—GDF-15	PA/AA-ratio ≥ 0.80	0.660	0.578–0.742	< 0.001	0.57	0.70	0.27
BNP—cTnI—suPAR	PA/AA-ratio ≥ 0.80	0.697	0.569–0.826	0.007	0.61	0.75	0.36
BNP—cTnI—IGF-BP2	PA/AA-ratio ≥ 0.80	0.715	0.588–0.843	0.004	0.63	0.74	0.37
BNP—cTnI—H-FABP	PA/AA-ratio ≥ 0.80	0.676	0.543–0.810	0.017	0.56	0.79	0.35
BNP—cTnI—GDF-15	PA/AA-ratio ≥ 0.80	0.708	0.576–0.840	0.005	0.50	0.93	0.43
BNP—cTnI—sST2	PA/AA-ratio ≥ 0.80	0.693	0.565–0.821	0.008	0.58	0.75	0.33

*BNP* brain natriuretic peptide, *cTnI* cardiac Troponin I, *sST2* soluble suppression of tumorigenicity-2, *GDF-15* growth/differentiation of factor-15, *H-FABP* heart-type fatty-acid binding protein, *IGF-BP2* insulin like growth factor binding protein 2, *suPAR* soluble urokinase-type plasminogen activator receptor

The best results in the 2-way biomarker analysis were obtained in the combination of cTnI and GDF-15 (AUC 0.710; 95% CI 0.588–0.832; YI 0.42; sensitivity 0.69; specificity 0.73; p = 0.002). The 3-way biomarker analysis was promising when combining cTnI, GDF-15 and IGF-BP2 (AUC 0.724; 95% CI 0.603–0.845; YI 0.43; sensitivity 0.71; specificity 0.72; p = 0.001).

## Discussion

### PA/AA-ratio: prognostic factor regarding 1-year survival

In the current clinical setting, diagnosis of PH as a sequelae of severe AS is mainly performed by TTE by determination of TRVmax or sPAP. However, not only echocardiography offers a noninvasive way of PH detection, but also computed tomography. In the context of procedure-planning CTA before TAVR, the main pulmonary artery diameter with a cut-off value of 29 mm and the PA/AA-ratio with a cut-off value of 1.00 (2015) and 0.90 (2022), respectively, found their way into the ESC guidelines so far [12, 13]. Recently, it could be shown in a publication of our group that in case of an echocardiographically obtained sPAP ≥ 40 mmHg, the cut-off value of the main pulmonary artery diameter is in accordance with the ESC guidelines. However, the main pulmonary artery parameter was not very conclusive in terms of mortality rates and also in terms of agreement with the expression of cardiovascular biomarkers [11]. The PA/AA-ratio had a very similar AUC-value (AUC 0.673; 95% CI 0.590–0.797; p < 0.001) as the main pulmonary artery diameter (AUC 0.676; 95% CI 0.580–0.771; p = 0.001) with respect to prediction of PH when sPAP ≥ 40 mmHg. Almost identical AUROC analyses were previously shown by Eberhard et al. [22], who calculated AUC-values of 0.63 for both main pulmonary artery diameter and PA/AA-ratio in a cohort of 257 TAVR patients using right heart catheterization data and a mean pulmonary artery pressure (mPAP) ≥ 25 mmHg as criteria for PH. However, in contrast to the study by Eberhardt et al., the cut-off value for PA/AA-ratio calculated in this study was shown to be an independent prognostic factor for long-term survival after TAVR and should possibly be included in clinical considerations regarding an eventually, conservative approach. Different sPAP cut-off values (40–45–50 mmHg) were clearly inferior to the PA/AA ratio in this study in terms of 1-year survival after TAVR.

### PA/AA-ratio  ≥ 0.80: potential for a new threshold?

The current ESC guidelines of August 2022 [13] state a PA/AA-ratio of 0.90 as a potential threshold for the presence of PH. This is already contrasted by a paper by O'Sullivan et al. [23] with a study of 139 TAVR patients, using right heart catheterization data and multi-detector computed tomography derived pulmonary vessel measurements, where an optimal cut-off value for the presence of PH was set at a PA/AA-ratio of 0.80. The AUC-value was 0.74 (95% CI 0.65–0.83; p < 0.001) with a sensitivity of 56% and a specificity of 88%. The cut-off value of 0.80 was consistently confirmed in our work, as this same value could be verified even for different sPAP values between 40–50 mmHg. The sensitivities and specificities amounted to values of 78% and 51%, which is most likely due to the more accurate examination methodology of right heart catheterization compared to TTE. Causes for the deviation from the ESC limit have already been argued by O'Sullivan et al. We, too, can confirm a generalized enlargement of the AA in the present collective with a mean age of 82.8 ± 4.9, as 49.5% had an AA ≥ 35 mm and 14.9% an AA ≥ 40 mm (data not shown). Pathophysiologically, changes in the structure of the collagenous and elastic fibers of the aorta are present, among others, leading to increased rigidity and a decrease in “Windkessel “ function. This rigidity, in turn, causes the development of arterial hypertension, which is present in a large proportion of TAVR patients, but is also causative of the structural changes in the elastic fibers of the AA. This occurring circulus vitiosus thus describes why in the elderly, and thus also in TAVR collectives, the PA/AA-ratio is less than in other PH collectives. Therefore, the cut-off value of PA/AA-ratio  ≥ 0.80 in elderly patient collectives should be preferred to the ESC reference values with ≥ 1.00 (2015) or 0.90 (2022).

### PA/AA-ratio: to what extent can biomarkers support the diagnosis?

Due to only 51% specificity of PA/AA-ratio for PH at sPAP ≥ 40 mmHg or to 53% at TRVmax ≥ 3.4 m/s, the aim of the study was to additionally support or optimize the diagnosis by common cardiovascular biomarkers. Comparative studies combining radiological criteria of PH with laboratory chemical markers are practically scarce. One of few papers was published by the working group of Gumauskiene et al. [24], who demonstrated that TAVR patients with an elevated sPAP (defined here ≥ 45 mmHg) had significantly higher BNP as well as GDF-15 values than TAVR patients with an sPAP < 45 mmHg. This result could be applied to our study results, because an sPAP ≥ 40 mmHg is associated with a PA/AA-ratio of ≥ 0.80, and in a group smaller or larger than the PA/AA-ratio cut-off value, respectively, equally significantly different expressions for these two cardiovascular biomarkers could be revealed.

**Fig. 12:**
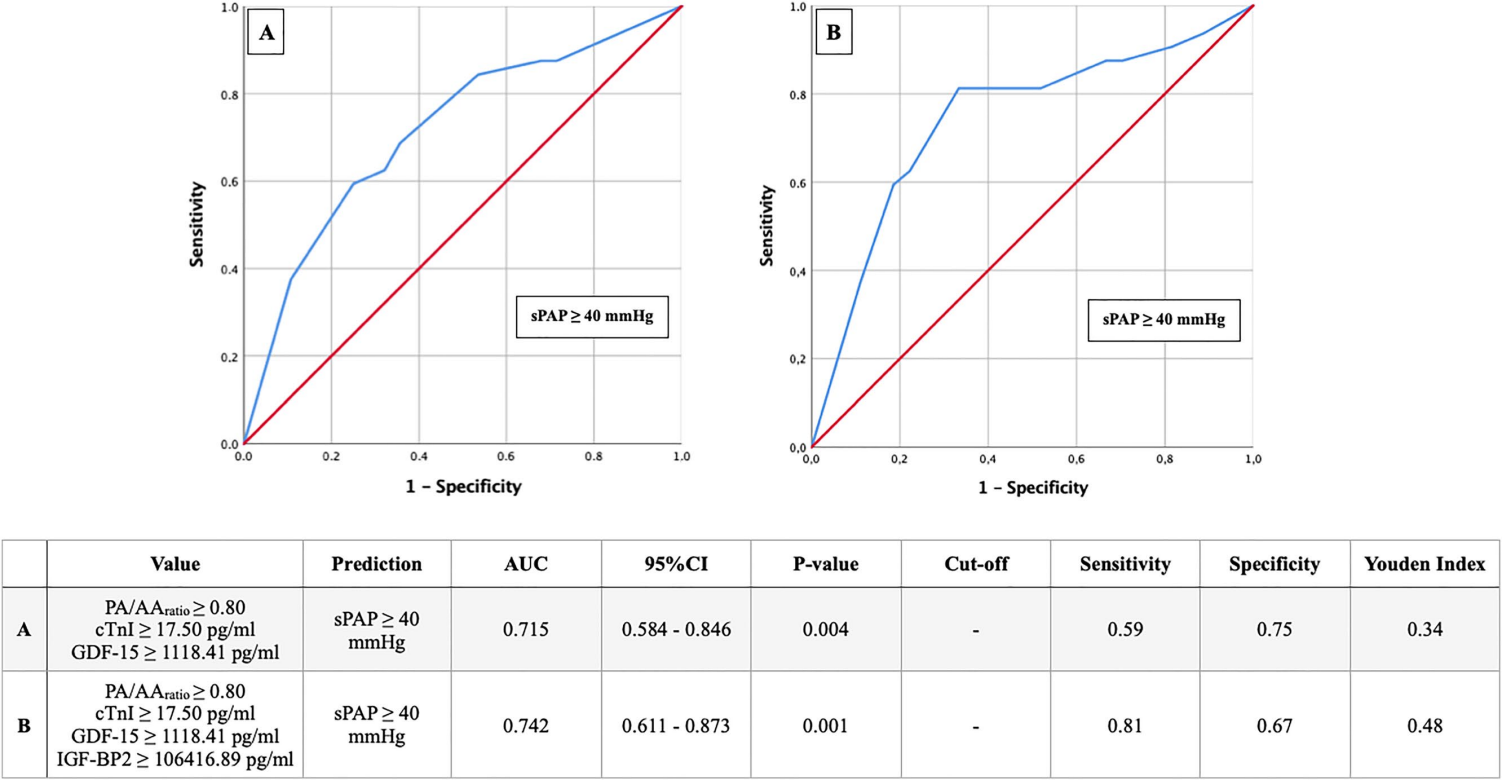
AUROC analyses of PA/AA-ratio with cardiovascular biomarker cut-off values for prediction of sPAP ≥ 40 mmHg with concerning cut-off values, Youden Index, sensitivity and specificity

In contrast to PA/AA-ratio, the main pulmonary artery diameter could not contribute to an early detection of pulmonary hypertension in a recently published study of our own working group [11], even in combination with cardiovascular biomarkers. By combined use of sPAP ≥ 40 mmHg, PA/AA-ratio  ≥ 0.80, a cTnI ≥ 17.50 pg/ml, GDF-15 ≥ 1118.41 pg/ml, and IGF-BP2 ≥ 106,416.89 pg/ml, however, the sensitivity for the presence of PH could be increased from 78 to 81% and the specificity from 51 to 67% (Fig. 12).

Finally, the noninvasive, radiological determination of PA/AA-ratio  ≥ 0.80 provides a diagnostic tool that can not only provide valuable information regarding 1-year mortality after TAVR but can also further delineate the risk for pulmonary hypertension with common cardiovascular biomarkers such as cTnI or GDF-15.

**Fig. 13:**
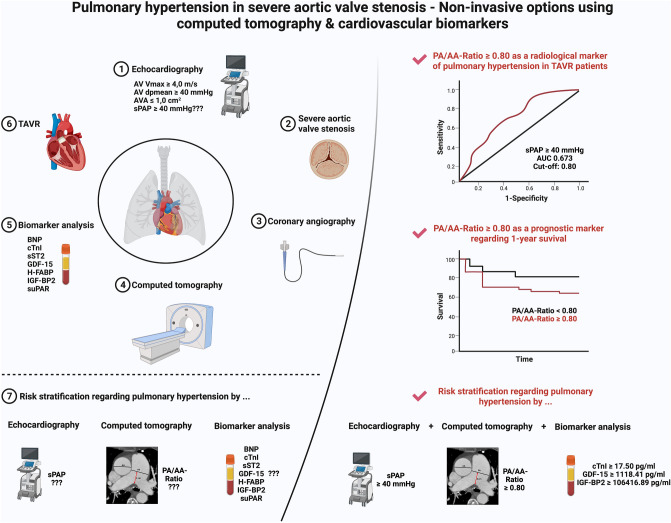
Graphical abstract of the study (created with BioRender.com)

A summary of the study design and the results obtained is presented in a graphical abstract in Fig. 13.

## Limitations

The present, retrospective study design is based on data from a small cohort over a circumscribed time period (2016–2018). Biomarker levels were only measured at baseline without statement regarding expression after TAVR procedure. Technical pitfalls in echocardiographic and radiological measurements which lead to misclassifications should always be conceded, even if examinations were performed by experienced clinical investigators. Additionally, invasive right heart catheterization, the gold standard of for accurate diagnosis regarding the genesis of PH (pre-capillary vs. post-capillary) was neither performed in Salzburg nor in Linz, because it is no longer a routine, diagnostic procedure before TAVR, since the findings are not a contraindication for TAVR and thus the mostly elderly and multi-morbid patients experience more risks than actual benefits from the invasive procedure. This final point also addresses the fact that, despite exclusion of obvious factors for pre-capillary pulmonary hypertension (CTEPH, PAH, interstitial lung disease, or underlying rheumatologic diseases with pulmonary involvement), we did not include with absolute certainty a pure cohort of only left heart-related, post-capillary pulmonary hypertension patients and thus isolated pre-capillary, but also combined pre-capillary and post-capillary patients, may also be found in this noninvasive study.

## Conclusion

With a PA/AA-ratio  ≥ 0.80, an underlying, quick and easily measurable radiological parameter can provide information about mortality in patients undergoing TAVR. The excellent inter- and intra-reader agreement (ICC > 0.98) for CT-measured diameters underlines the reproducibility and robustness of this ratio. Combination of CT, TTE and cardiovascular biomarkers offers a potential way of noninvasive risk stratification regarding pulmonary hypertension in patients with severe aortic valve stenosis.

## Data Availability

The datasets generated and analysed during the current study are available from the corresponding author on reasonable request.
